# International Standards for Dementia Workforce Education and Training: A Scoping Review

**DOI:** 10.1093/geront/gnad023

**Published:** 2023-04-18

**Authors:** Sabrina Winona Pit, Louise Horstmanshof, Anne Moehead, Oliver Hayes, Valerie Schache, Lynne Parkinson

**Affiliations:** University Centre for Rural Health, The University of Sydney, Lismore, New South Wales, Australia; School of Medicine, Western Sydney University, Lismore, New South Wales, Australia; Southern Cross University, Lismore, New South Wales, Australia; Dementia Inclusive Ballina, Ballina, New South Wales, Australia; University of Melbourne, Melbourne, Victoria, Australia; Dementia Alliance International, Ballina, New South Wales, Australia; School of Medicine and Public Health, The University of Newcastle, New South Wales, Australia; Olivia May Consulting, Gladstone, Queensland, Australia

**Keywords:** Aged care, Education, Policy, Standards, Workforce

## Abstract

**Background and Objectives:**

The increasing number of people with dementia requires transparency and quality dementia education, training, and care. This scoping review aimed to determine the key elements of national or state-wide standards on dementia education and training that could underpin the development of international standards for dementia workforce training and education.

**Research Design and Methods:**

The English-language peer-reviewed and gray literature were searched (2010–20). Key search domains were training, workforce, standards/frameworks, and dementia.

**Results:**

Thirteen standards were identified from the United Kingdom (*n* = 5), the United States (*n* = 4), Australia (*n* = 3), and Ireland (*n* = 1). Most standards focused on training health care professionals with some including people in customer-centric settings, people living with dementia, and informal carers or the general community. Seventeen training topics were identified in 10 or more of the 13 standards. Cultural safety, rural issues, health care professional self-care, digital literacy, and health promotion topics were less commonly reported. The barriers to standards implementation were lack of organizational support, lack of access to relevant training, low staff literacy, lack of funding, high staff turnover, ineffective past program cycles, and inconsistent service delivery. Enablers included a strong implementation plan, funding, strength of partnerships, and building on previous work.

**Discussion and Implications:**

The U.K. Dementia Skills and Core Training Standard, the Irish Department of Health Dementia Together, and the National Health Services Scotland Standard are the recommended strongest standards for underpinning the development of international standards. It is essential that training standards are tailored to the needs of the consumer, worker, and regions.

## Background and Objectives

In 2020, it was estimated that worldwide 50 million people were living with dementia. This figure is expected to rise to 152 million people by 2050 (Alzheimer’s Disease International, 2020b). National dementia policy and action plans are evolving globally to deal with the rise in dementia. Alzheimer’s Disease International (ADI) reports that in 2021, only 30 out of 194 countries/territories have existing national dementia plans, 21 are developing a plan, 2 countries (Russia and Finland) have integrated dementia plans under other policy areas, whereas 141 countries or territories have no plan (Alzheimer’s Disease International, 2021a). Education and training form part of the World Health Organization (WHO)’s “Global action plan on the public health response to dementia 2017–2025”; a dementia education and training standard can guide the development of education and training programs (WHO, 2017).

The rise in dementia calls for a highly educated and skilled dementia care workforce globally. Informal carers form an essential part of this workforce. In 2020, the estimated total cost to replace the Australian informal care workforce with paid carers equated to $USD 56.7 billion (Deloitte Access Economics, 2020). In 2020, there were over 2.8 million carers and 906,000 primary informal carers. It was estimated that primary informal carers provide on average 35.2 care hours per week, which equals to about 2.2 billion hours of unpaid care in 2020 (Deloitte Access Economics, 2020). Between 2020 and 2030, the demand for informal carers is expected to increase by 23% (1.25–1.54 million) whereas the supply of carers is expected to decrease by 16% (674,000–780,000; Deloitte Access Economics, 2020). These figures suggest that increasing pressure will be placed on the dementia workforce in years to come. Similar pressures and high costs are found across the globe with the annual global cost of dementia care exceeding $USD 1 trillion. This cost can approximately be attributed to informal care (40%), social care (40%), and medical care (20%), with informal care costs being highest in African regions and social care being highest in some South American regions, North America, and Western Europe (Alzheimer’s Disease International, 2021a).

There is a growing recognition internationally of the need for high-quality and specialized dementia care training and education that meets quality and safety standards. A 2018 systematic review of 26 National Dementia Strategies identified that improved education and training for health care professionals was a priority in 16 countries, including Australia, Cuba, Czech Republic, England, Greece, Indonesia, Ireland, Israel, Italy, Korea, Malta, Mexico, Puerto Rico, Switzerland, the United States, and Wales (Chow et al., 2018). The focus of training differed by country, for example, Ireland and Israel, concentrated their key action items on diagnosis and management of dementia care for general practitioners while Cuba, the Czech Republic, Mexico, Korea, and the United States wanted to increase the number of dementia care specialists. Greece planned to offer scholarships and Cuba planned to offer refresher dementia courses for health care professionals. Chow et al. (2018) focused on national dementia strategies, whereas this study focused solely on education and training and aimed to support national dementia strategic plans in the area of education and training programs.

Although some workforce training and education solutions have already been implemented (DeSouza et al., 2020; Goldberg et al., 2015), literature reviews suggest that the implementation of programs varies widely across the globe (Abley et al., 2019; Alushi et al., 2015; Moehead et al., 2020). Hvalič-Touzery et al. (2018) explored the level of dementia care taught at accredited European higher education providers of undergraduate and postgraduate levels, studies in nursing, medicine, psychology, social work, physiotherapy, occupational therapy, and gerontology. There was a lack of a dementia focus in undergraduate health and social care programs, and study programs were highly variable across countries and education levels. Countries such as Singapore have specialized training courses for foreign domestic workers who assist the aging Singaporean population (Alzheimer’s Disease Association Singapore, 2020) and other countries have implemented dementia awareness training, for example, by providing toolkits for general businesses to educate people at the frontline (Dementia Australia, 2019) such as shop assistants and bank tellers or the community at large. Given the complexity and inconsistency around dementia education and training globally, an overview of existing international education and training standards for dementia care training could potentially be helpful. An overview of these standards can inform countries that have yet to develop such a standard and guide the development of an international standard.

Some international organizations are already working towards standards in dementia care training and education. The International Organization for Standardization established a Technical Committee on Aging Societies in 2018 and has published its first three international standards including Age-Inclusive Workforce, Dementia-Inclusive Communities, and Carer Inclusive Organizations (Pit, Livingstone, et al., 2022). The age-inclusive workforce international standard (International Organization for Standardization, 2022a) has a guideline section on education and how to work with people living with dementia in the workplace. And, the dementia inclusive communities international standard recommends training of both informal and formal caregivers to improve care and public education to raise awareness (International Organization for Standardization, 2022b). ADI has established accreditation standards and criteria to assist dementia care providers, train-the-trainers, health care professionals, home care workers, and family caregivers (Alzheimer’s Disease International, 2020a) in developing high-quality education programs. These standards provide information on the important questions that should be asked about all aspects of a training program.

More detailed guidance is needed to improve dementia education and training. There is a clear opportunity to develop an international standard for dementia education and training. To determine whether this is feasible, this study aims to explore and compare the key content of available standards that could underpin the development of international standards for workforce dementia education and training, and also inform countries that have yet to develop a national dementia education and training standard. The research question was: “What are the key elements covered in available national and state-wide standards on dementia education and training that could underpin the development of international standards for dementia workforce education and training?” The results will be useful for all people involved in standards development, including peak governing bodies, industry associations and experts, academics, accreditation bodies, consumer and carer organizations, and policy-makers.

## Research Design and Methods

The study was informed by funded policy review work which sought to determine which standards frameworks would be useful for developing a standard within Australia (Pit, Horstmanshof, et al., 2022).

### Eligibility Criteria

Focus: Describes the development of dementia care education or training standards. For this study, “standards” was defined as frameworks, guidelines, specifications, benchmarks, and requirements.

Location: All countries but focusing on English-speaking and English-language articles.

Settings: Education/training for those providing care and services for people with dementia in the community, primary health care, emergency departments, inpatient care, and aged care facilities.

Population: Education/training for people who are part of a paid workforce such as nurses, doctors, social workers, and care workers who deliver care or services for people with dementia.

Time frame: Last 10 years.

Type of item: All relevant items (including websites, reports, and presentations).

### Information Sources

A quasi-systematic search of electronic databases including OVID Medline, Embase, CINAHL Complete, ERIC, PsycINFO, and Cochrane was undertaken. Gray sources included Google search engine, Opengrey, LMNbookshelf, Health Sciences Online, Analysis and Policy Observatory, MedNar, Science.gov, OIAster, OpenDOAR, WorldWideScience, and relevant government websites, Alzheimer’s and dementia organization websites, and disciplinary colleges and organizations. Items from January 2010 to December 2020 were searched. Searches were conducted in December 2020.

### Search

The full electronic search strategy is available in Section 1 of Supplementary Material.

### Selection of Sources of Evidence

Searches and initial screening by title and abstract were undertaken by L. Parkinson, S. W. Pit, L. Horstmanshof, O. Hayes, and A. Moehead. Screening of full texts was undertaken by L. Parkinson, S. W. Pit, L. Horstmanshof, O. Hayes, and A. Moehead (using an exchange of results between reviewers, to ensure at least two reviewers considered each item).

### Data Charting Process

Item references were stored in Endnote. A standardized, pilot-tested Excel spreadsheet was used to extract data from items. Data extraction was undertaken by all reviewers. Each reviewer appraised and extracted data for the items identified by another reviewer. At least two reviewers checked data extraction.

### Data Items

The data items collated were Author/Institution; Title; Year published; Country; Setting (community, primary health care, emergency departments, inpatient care, and aged care facilities); Standard principles and values; Training topics; Key sections of standards; Standard development; Consumer input to development; Challenges, enablers, and barriers to development and implementation; Implementation of the standard (Yes or No); Evaluation of the standard (Yes or No); Conclusions, and Recommendations if any.

### Critical Appraisal of Individual Sources of Evidence

Risk of bias was not relevant to the items of interest for our research question as they were mostly not scholarly articles; however, the Australian National Health and Medical Research Council (NHMRC) levels of evidence were used as a guideline for the strength of the evidence base for each standard (NHMRC, 2009). This guideline designates evidence base as A = excellent—several Level I or II studies with low risk of bias; B = good—one or two Level II studies with low risk of bias or a systematic review or multiple Level III studies with a low risk of bias; C = satisfactory—Level III studies with low risk of bias, or Level I or II studies with moderate risk of bias; D = poor—Level IV studies, or Level I–III studies/systematic reviews with a high risk of bias; √ = best practice.

Levels of evidence include: I = a systematic review of Level II studies; II = a randomized controlled trial; III-1 = a pseudo-randomized controlled trial (i.e., alternate allocation or some other; method); III-2 = a comparative study with concurrent controls (i.e., nonrandomized experimental trials, cohort studies, case–control studies, interrupted time series studies with a control group); III-3 = a comparative study without concurrent controls (i.e., historical control study, two or more single-arm studies, interrupted time series studies without a parallel control group); IV = case series with either post-test or pretest/post-test outcomes (NHMRC, 2009).

### Synthesis of Results

An inductive narrative thematic approach was used for item description and summary. We did not have an a priori view of what key sections should be included. The focus of the analysis was the standard content; challenges to developing and implementing standards; enablers that have supported development and implementation; and level of engagement with stakeholders. At least two reviewers each read through the extracted data and identified and recorded commonalities and differences between the standards in an excel spreadsheet. All data were checked by at least one other reviewer. These spreadsheets then formed the basis for further synthesis and analyses. Tabulated summaries were created to compare and contrast the themes arising, which were verified by at least one other reviewer. Discrepancies between reviewers at every stage were resolved through discussion and consensus. The final verified themes and categories are presented in tables to enable ease of interpretation for the reader (Levac et al., 2015).

## Results

### Selection of Sources of Evidence

**Figure 1.**
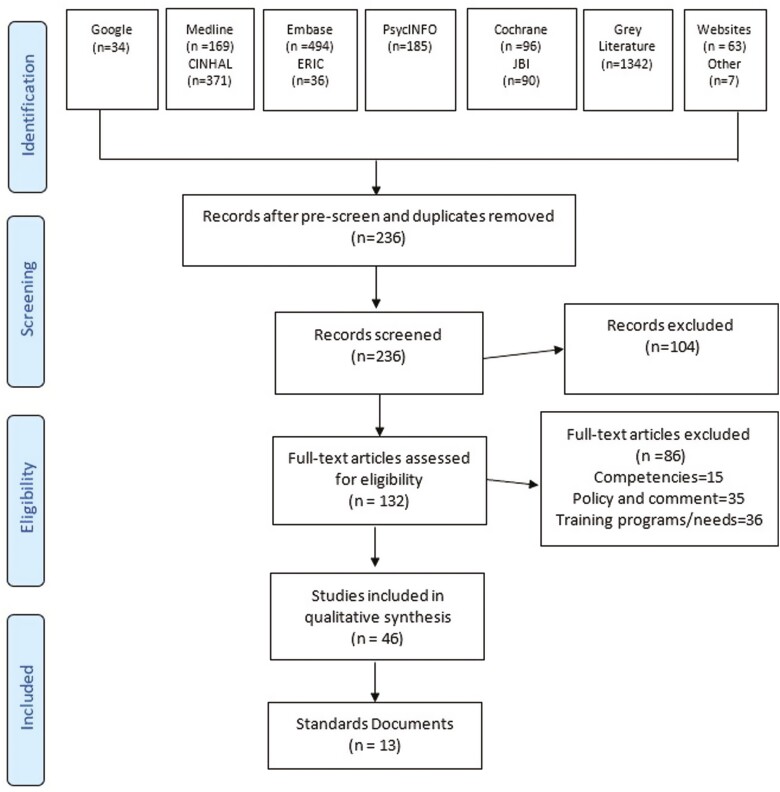
Flow diagram selected international standards [adapted from Pit, Horstmanshof, et al. (2022) and Moher et al. (2009)].

Figure 1 details the selection of items for the review. From a potential 2,887 items, 236 items remained after prescreen and duplicate removal; 132 full-text items were assessed for eligibility; 46 items were included in the qualitative synthesis; and 13 standards were identified.

### Characteristics of Sources of Evidence

#### Country and region.

The 13 identified standards originated from Australia (*n* = 3; one national, two state level from Queensland; Australian Primary Health Care Nurses Association, 2015; Queensland Health, 2010, 2020), United Kingdom (*n* = 5; four national level: United Kingdom, Northern Ireland, Scotland, and Wales and one regional Scotland; Care Council for Wales, 2016; Dementia Together Northern Ireland, 2016; NHS Greater Glasgow & Clyde Dementia Services Workforce Development Group [DWDG], 2012; Skills for Care and Skills for Health, 2011; Skills for Health and Health Education England and Skills for Care, 2015, 2018; The Scottish Government, 2011), United States (*n* = 4; two national and two state level from Georgia and West Virginia; Alzheimer’s Association and Centers for Disease Control and Prevention, 2018; Georgia Alzheimer’s and Related Dementias Collaborative Workforce Development Committee, 2016; Newbrough, 2011; US Department of Health and Human Services, 2019), and Ireland (*n* = 1; national level; Department of Health Ireland, 2014, see Table 1).

**Table 1.** Characteristics of Standards: Country, Region, and Focus Workforce

Country	Region	Workforce	Source	ID
Australia	National	Primary Healthcare Nursing	Australian Primary Health Care Nurses Association (2015)	S1
Australia	Queensland	Government Health Staff	Queensland Health (2010)	S2
Australia	Queensland	People involved in end-of-life care	Queensland Health (2020)	S3
Ireland	National	All care staff	Department of Health Ireland (2014)	S4
United Kingdom	National	Health and care workforce	Skills for Care and Skills for Health (2011); Skills for Health and Health Education England and Skills for Care (2015); Skills for Health and Health Education England and Skills for Care (2018); UK National Institute for Health Care Excellence (2018)	S5
United Kingdom: Northern Ireland	National	Health and social care staff	Dementia Together Northern Ireland (2016)	S6
United Kingdom: Scotland	Greater Glasgow and Clyde	Health and social care staff	NHS Greater Glasgow & Clyde Dementia Services Workforce Development Group (2012)	S7
United Kingdom: Scotland	National	Health and social care staff	The Scottish Government (2011)	S8
United Kingdom: Wales	National	Health and social care staff	Care Council for Wales (2016)	S9
United States	National	Public Health staff	Alzheimer’s Association and Centers for Disease Control and Prevention (2018)	S10
United States	National	Health care staff across the care continuum	US Department of Health and Human Services (2019)	S11
United States	Georgia	Direct care workers	Georgia Alzheimer’s and Related Dementias Collaborative Workforce Development Committee (2016)	S12
United States	West Virginia	Health professionals, direct care workers, and informal caregivers	Newbrough and Boone (2011)	S13

#### Workforce populations.

All five U.K.-based standards focused on health and social care staff (UK National Institute for Health Care Excellence, 2018). Australian standards focused on primary health care nursing (Australian Primary Health Care Nurses Association, 2015), QLD government health staff (Queensland Health, 2010), and QLD-based end-of-life care workforce (Queensland Health, 2020). Two standards in the United States focused on national public health staff and health care staff (Alzheimer’s Association and Centers for Disease Control and Prevention, 2018; US Department of Health and Human Services, 2019), the Georgia-state standard (Georgia Alzheimer’s and Related Dementias Collaborative Workforce Development Committee, 2016) only included direct care workers, whereas West Virginia (Newbrough, 2011) included health professionals, direct care workers, and informal caregivers. Some standards mentioned that other audiences may benefit from using the standard from a training perspective such as training providers (Alzheimer’s Association and Centers for Disease Control and Prevention, 2018; Dementia Together Northern Ireland, 2016; Queensland Health, 2020; The Scottish Government, 2011), customer-focused work settings such as banks and shops and faith-based groups (Alzheimer’s Association and Centers for Disease Control and Prevention, 2018; Care Council for Wales, 2016; Queensland Health, 2020; UK National Institute for Health Care Excellence, 2018), and people living with dementia and their carers (Care Council for Wales, 2016; Department of Health Ireland, 2014; Newbrough, 2011; Queensland Health, 2020; The Scottish Government, 2011; US Department of Health and Human Services, 2019).

### Critical Appraisal Within Sources of Evidence

The identified standards were built on previous work, existing policies, and reviews of existing resources and literature. Consumer input from people living with dementia and their carers was canvassed in 6 out of 13 standards (Alzheimer’s Association and Centers for Disease Control and Prevention, 2018; Australian Primary Health Care Nurses Association, 2015; Care Council for Wales, 2016; Dementia Together Northern Ireland, 2016; The Scottish Government, 2011; UK National Institute for Health Care Excellence, 2018). Stakeholders included Alzheimer’s societies, government agencies, university academics, and nonprofit organizations involved with care of people living with dementia and their carers and families. The authors graded the body of evidence for S5 (UK National Institute for Health Care Excellence, 2018) and S8 (The Scottish Government, 2011) as B; S4 (Department of Health Ireland, 2014), S6 (Dementia Together Northern Ireland, 2016), and S7 (NHS Greater Glasgow & Clyde Dementia Services Workforce Development Group (DWDG), 2012) were graded as C by Australian NHMRC standards (NHMRC, 2009). The remaining standards relied on recommended best practice and policy.

### Content of Individual Sources of Evidence

#### Principles and values.

Eighteen themes around principles and values were identified. Table 2 presents the most common themes included in the standards: community (*n* = 13) and workforce and services (*n* = 13), followed by support (*n* = 12), then early diagnosis, information provision, quality of care, and choice (*n* = 10). The Wales (Care Council for Wales, 2016) Standard included all 18 themes identified, followed by the United Kingdom with 17 (UK National Institute for Health Care Excellence, 2018). Examples of principles and values are as follows:

**Table 2.** Most Common Themes Across Standard Principles and Values

ID	Community involvement	Appropriately trained and skilled workforce with tailored service delivery	Support for people living with dementia, their carers, and the workforce	Early diagnosis	Information provision and access	Quality of care	Choice
S1	✓	✓	✓	✓	✓	✓	✓
S2	✓	✓	✓	✓	✓		✓
S3	✓	✓	✓		✓	✓	✓
S4	✓	✓	✓	✓			✓
S5	✓	✓	✓	✓	✓	✓	✓
S6	✓	✓	✓	✓	✓	✓	✓
S7	✓	✓	✓		✓	✓	
S8	✓	✓	✓	✓	✓		✓
S9	✓	✓	✓	✓	✓	✓	✓
S10	✓	✓	✓	✓	✓	✓	✓
S11	✓	✓	✓	✓		✓	
S12	✓	✓	✓			✓	✓
S13	✓	✓		✓	✓	✓	

*Note*: ✓ = included in standard.

- Community: “Training activities should be coordinated among key training partners (such as universities, community and technical colleges, adult learning centers, long-term care facilities, senior centers, and the Alzheimer’s Association) to make certain that the competency needs of all three workforce sectors (health professionals, direct care workers, and informal caregivers) are met” (S12).
- Workforce and services: “To receive safe care and treatment from staff who are suitably qualified, competent and well-motivated to undertake their roles” (S6).
- Support: “The importance of taking account of the needs of carers (whether they are family and friends or paid care workers), and supporting and enhancing their input” (S5).
- Early diagnosis: “A person-centred approach that includes: The promotion of healthy aging strategies across the life span; Earlier screening, diagnosis and/or referral to specialist services” (S2).
- Information on provision: “Receive information and the necessary support they need to continue to participate in decisions which affect them now and in the future” (S8).
- Quality of care: “A well-trained supported workforce that delivers quality care” (S3).
- Choice: “People have the right to make decisions that others may think unwise” (S9).

#### Training/education topics.

Fifty-five topics were identified overall. Table 3 presents the topic themes that were included in the standards in order of most commonly reported. Topics were clustered by care setting, basic skills, advanced skills, health promotion, ethics and values, and staff support. Staff support had the lowest identified training topic areas, with the exception of leadership in dementia care (*n* = 10) and health professional self-care and health literacy being the lowest (*n* = 1). Digital health literacy was listed in six standards. Northern Ireland (Dementia Together Northern Ireland, 2016), Scotland (The Scottish Government, 2011), and Wales (Care Council for Wales, 2016) standards included the most topics. The most common topics were people in regular close contact with people with dementia (*n* = 13), basic dementia awareness (*n* = 12), communication in dementia care (*n* = 12), community care (*n* = 12), recognizing delirium (*n* = 12), and understanding legal issues and legislation (*n* = 12).

**Table 3.** Training Topic Themes Included in Standards

Training topics	Care setting	Basic skills	Advanced skills	Health promotion	Ethics and values	Staff support	Number of standards listed
People in regular close contact with people living with dementia	✓						13
Basic dementia awareness		✓					12
Communication in dementia care		✓					12
Community care	✓						12
Recognizing delirium		✓					12
Understanding legal issues and legislation					✓		12
Dementia risk reduction and prevention		✓					11
Diagnosis of dementia			✓				11
End-of-life care for people living with dementia			✓				11
Ethics, potential risks, and safeguards					✓		11
Home care	✓						11
Hospital and acute care	✓						11
Evidence-based decisions and practice		✓					10
Nonpharmacological behavior management			✓				10
Maintaining well-being (all categories)^a^				✓			10
Palliative care			✓				10
Leadership in dementia care						✓	10
Primary health	✓						9
Communication with carer or family		✓					9
Maintaining well-being: social				✓			9
Enabling environment				✓			9
Recording and reporting					✓		9
Equality, diversity, and inclusion (all categories)^b^					✓		9
Integrated care	✓						8
Person-centered care		✓					8
Communication with people living with dementia		✓					8
Nutrition/food/oral health		✓					8
Younger onset dementia			✓				8
Advance care planning/directive/living will			✓				8
Maintaining well-being: psychological				✓			8
Local nonmedical support (including advocacy)						✓	8
Rehabilitation	✓						7
Residential aged care/nursing home	✓						7
Holistic care		✓					7
Cognitive assessment and screening/memory function			✓				7
Pharmacological care			✓				7
Assistive technology				✓			7
Biopsychosocial assessment			✓				6
Pain management, including effect on people living with dementia			✓				6
Maintaining well-being: spiritual				✓			6
Digital/health literacy						✓	6
Maintaining well-being: physical (including comorbidities)				✓			5
Maintaining carer well-being				✓			5
Equality, diversity, and inclusion: cultural and linguistic backgrounds					✓		5
Understand dementia care funding						✓	5
Maintaining well-being: mental (including comorbidities)				✓			4
Prevention of falls and fractures				✓			4
Equality, diversity, and inclusion: First Nations Peoples					✓		4
Navigating the aged care support system						✓	4
Respite care	✓						3
Telehealth						✓	2
Maintaining well-being: employment				✓			1
Housing				✓			1
Health literacy						✓	1
Health professional self-care						✓	1
Trauma informed care			✓				0
Equality, diversity, and inclusion: rural and remote							0

^a^Maintaining well-being for people living with dementia subcategories: physical (including comorbidities), mental (including comorbidities), psychological, social, spiritual, and employment.

^b^Equality, diversity, and inclusion subcategories: First Nations Peoples, cultural and linguistic backgrounds, rural, and remote.

#### Key sections of standards:

Table 4 details the key sections included in at least five of the identified standards. Section 2 of Supplementary Material includes details of all sections included across all standards. Evidence underpinning the standard, target audience, purpose and principles underpinning the document, and thematic subjects or key priority areas (topics) were included in all identified standards. An evaluation plan, indicators (or outcome measures or success factors or skills statements), and tiers or levels of practice or training/education were included in 8 of the 13 standards; an implementation plan, strategies, actions or recommendations, and structure of document were detailed in 7 of the 13 standards; and how to use the document, and links to relevant training resources (e.g., online modules) were included in six of the standards.

**Table 4.** Key Sections of Standards (Included in Five Standards or More)

Content	Standards including section
Evidence supporting the standard (e.g., policy and research)	All
Purpose and guiding principles	All
Target audience	All
Topics or priority areas	All
Evaluation plan	S2, S3, S4, S5, S8, S9, S10, S11
Indicators, outcome measures, success factors, and skills statements	S1, S2, S3, S5, S8, S10, S12, S13
Tiers or levels of practice or training	S3, S4, S5, S6, S7, S8, S9, S13
Implementation plan	S1, S2, S3, S4, S5, S7, S8
Strategies, actions, or recommendations	S1, S2, S4, S5, S7, S10, S11
Structure of document	S1, S2, S3, S4, S5, S6, S8
Explanation of how to use the document	S5, S7, S8, S9, S10, S12
Links to appropriate training resources	S3, S5, S7, S9, S12, S13

### Consumer and Other Stakeholder Participation, Barriers, and Enablers to Development and Implementation

#### Engagement with consumers and stakeholders.

Five standards described good levels of consumer input (Dementia Together Northern Ireland, 2016; Department of Health Ireland, 2014; Georgia Alzheimer’s and Related Dementias Collaborative Workforce Development Committee, 2016; The Scottish Government, 2011; UK National Institute for Health Care Excellence, 2018), but three standards did not mention any consumer input (Alzheimer’s Association and Centers for Disease Control and Prevention, 2018; Newbrough, 2011; Queensland Health, 2020). All standards included a focus on health care professionals, although only four standards included customer-centric work settings such as shops and banks (Alzheimer’s Association and Centers for Disease Control and Prevention, 2018; Care Council for Wales, 2016; Queensland Health, 2020; UK National Institute for Health Care Excellence, 2018). In particular, Wales (Care Council for Wales, 2016) used a very different implementation model to other standards, adopting the concept of influencers and promoting specific training for influencers. Influencers are dementia advocates or leaders who do not have to be health care professionals; they can be someone with lived experience, for example, early-onset dementia, who can raise the profile of dementia. Influencer learning topics focus on engagement.

Seven standards described good coverage of relevant stakeholder input (Alzheimer’s Association and Centers for Disease Control and Prevention, 2018; Dementia Together Northern Ireland, 2016; Department of Health Ireland, 2014; Georgia Alzheimer’s and Related Dementias Collaborative Workforce Development Committee, 2016; NHS Greater Glasgow & Clyde Dementia Services Workforce Development Group (DWDG), 2012; The Scottish Government, 2011; UK National Institute for Health Care Excellence, 2018); only S3 (Queensland Health, 2020) did not mention other stakeholder input, but did list their partners.

#### Enablers.

Table 5 outlines the reported enablers and barriers to the development and implementation of the standards. The most common enabler was building on past work (11 of 13 standards), followed by access to funding (10 of 13 standards), and strength of partnerships (9 of 13 standards). A strong plan for implementation was a particular enabler for implementation of a standard (7 of 13 standards). One standard (Newbrough, 2011) did not report any enablers.

**Table 5.** Barriers and Enablers to Development and Implementation of Standards

Enablers and barriers	S1	S2	S3	S4	S5	S6	S7	S8	S9	S10	S11	S12	S13
Enablers													
Building on past work	D	B	B	B	D	D	D		B	B	B	D	
Funding from government, grant, or other bodies	D	B		B	B	D	D		B	B	B	D	
Strength of partnerships				B	B	D	D	B	B	B	B	D	
Strong plan for implementation	I	I	I	I	I		I	I					
Strength of research including case studies				B	D	D	D	B					
Peak body endorsement	D	B			B							D	
Linked to mandatory accreditation			B										
Access to appropriate and relevant training					I								
Barriers													
Unsupportive rules and regulations					I			I	D				
Staff issues (literacy, roles, and turnover)	D				I								B
Lack of progress in past cycles				I						I			
Lack of organizational support					I			I					
Lack of appropriate and relevant training				I	I								
Lack of funding or cost of research					B								
Inconsistent services				I									

*Notes:* D = for development only, I = for implementation only, and B = for both.

#### Barriers.

Six of the 13 standards did not report any barriers to the development and implementation of the standards. The most common barriers reported were unsupportive rules and regulations, and staff issues (three of the seven standards reporting barriers).

### Synthesis of Results

Table 6 summarizes the level of content described across the 13 standards: principles and values, training topics, key sections, consumer and stakeholder input, and level of evidence underpinning the standard. The stand-out standard of this synthesis was the Scottish Standard (The Scottish Government, 2011) as it addressed all sections arising across the standards and included the most frequently incorporated items within each section. Four other standards covered four out of six elements (Dementia Together Northern Ireland, 2016; Department of Health Ireland, 2014; NHS Greater Glasgow & Clyde Dementia Services Workforce Development Group (DWDG), 2012; UK National Institute for Health Care Excellence, 2018).

**Table 6.** Synthesis of Standards by Content

ID	Principles and values	Training topics	Key sections	Consumer input	Stakeholder input	Evidence grade
	5 of top 5	12 of top 12	10 of top 10	Good	Good	B or C
S1	✓					
S2	✓					
S3			✓			
S4			✓	✓	✓	✓
S5	✓		✓		✓	✓
S6	✓			✓	✓	✓
S7		✓	✓		✓	✓
S8	✓	✓	✓	✓	✓	✓
S9	✓	✓		✓		
S10	✓	✓			✓	
S11						
S12				✓	✓	
S13		✓				

*Note* **:** ✓ = included in standard.

## Discussion and Implications

This scoping review was the first step to inform the development of an international standard for dementia education and training. It also provides guidance for country-specific dementia education and training standards. Thirteen standards, published between 2010 and 2020, were identified. Standards varied in the complexity of target audiences, values, learning topics, structural key content, consumer and stakeholder input, and development methodologies. Although this review examined an extensive list of the elements of available standards, none of the standards had all of the proposed elements. Countries that are developing a dementia education standard may use the lists of key elements and the identified best standards (Dementia Together Northern Ireland, 2016; Department of Health Ireland, 2014; NHS Greater Glasgow & Clyde Dementia Services Workforce Development Group (DWDG), 2012; The Scottish Government, 2011; UK National Institute for Health Care Excellence, 2018) as the bases for how to structure their standard. For an international standard, inclusion of key elements should be discussed with relevant global-level stakeholders.

### Principles and Value

Multiple principles and values were identified in the varying standards. Table 2 shows the top 10 themes for principles and values. The most common principles were the involvement of community, a focus on workforce and services (which were concepts within all standards), support, early diagnosis, and information provision. Only one standard, the Wales Standard (Care Council for Wales, 2016), included 18 principles and values. There is an increased application of using sustainable development goals to identify the value of international standards (Pit, Livingstone, et al., 2022), future standard developers could consider these goals to guide the development of their own principles and values when designing a dementia education and training international standard.

### Training and Education Topics

The 57 training/education topics identified, clustered by care setting, basic skills, advanced skills, health promotion, ethics and values, and staff support, can assist standard developers in identifying topic choices. The topics identified were broadly supported by the international literature (DeSouza et al., 2020; Traynor et al., 2011). The most common topics fell under basic skills training such as dementia awareness training, dementia risk reduction, communications, evidence-based practice, and delirium. Then care settings and advanced skills such as diagnosis, end-of-life care, palliative care, and nonpharmacological management. The hierarchy of topics mirrors some of the global concerns around the delay in diagnosing people with dementia and a call for improving dementia prevention through risk reduction (Alzheimer’s Disease International, 2021a). It is, therefore, pleasing to note that early diagnosis was covered in the values and principles in the majority of standards. However, few training topics covered health promotion areas such as housing (*n* = 1) and falls prevention (*n* = 4).

Notably, maintaining carer well-being is featured in only five standards. This is an oversight. Every training level should consider carer well-being—informal carers provide high value to society and large healthcare cost-savings for the government, often at the cost of lowering their own quality of life (Deloitte Access Economics, 2020). Staff support had the lowest identified training topic areas, with the exception of leadership in dementia care (*n* = 10). Although leadership training is important, training for operational workers is equally important to sustain their workforce capability (Moehead et al., 2020). Training for operational workers should include knowledge on dementia care funding to allow the maximum income to be generated for care facilities, telehealth, and an understanding of flected at the international how to navigate the complex aged care system. The most common training topics around ethics and values included understanding legal issues (*n* = 12) and ethics, risks, and safeguards (*n* = 11).

### Consumer Input and Dementia Person-Centered Design

A scoping review (Wang et al., 2019), looking at the global literature, identified that including people living with dementia in research design is valuable for both the people living with dementia and the design process. Furthermore, the Australian NHMRC suggests that there are clear benefits and documented evidence of the impact of consumer involvement on health and medical research. The NHMRC endorses that guideline developers “should actively seek to increase the levels of consumer involvement as much as possible throughout guideline development and to strive for equal and alike participation” (NHMRC, 2018). This is reflected at the international level where consumers form part of aging societies’ standards development process (Pit, Livingstone, et al., 2022). As international standards are often written in the form of guidelines, we argue that consumer participation and engagement in codesigning a standard is a potential enabler for implementation success, and was indeed reported as a success factor in 10 out of 13 standards. Training for people living with dementia and their carers was included in six standards (Care Council for Wales, 2016; Department of Health Ireland, 2014; Newbrough, 2011; Queensland Health, 2020; The Scottish Government, 2011; US Department of Health and Human Services, 2019). Based on our findings, to further translate consumer participation into practice the following standard elements can be considered when developing a dementia education standard to further strengthen the usefulness: foreword by an experienced expert, a person living with dementia (Dementia Together Northern Ireland, 2016), statements by people living with dementia (Dementia Together Northern Ireland, 2016; The Scottish Government, 2011), the use of patient journeys (Dementia Together Northern Ireland, 2016; The Scottish Government, 2011), and case studies demonstrating the application of the standards (Alzheimer’s Association and Centers for Disease Control and Prevention, 2018).

### Importance of Stakeholders

Engaging the community at large in standards development was not apparent in many standards. This contrasts with community being a principle or a value in all standards. The majority (9 out of 13) of the standards did not include customer-centric work settings such as shops and banks. Educating the wider public is necessary to enable change on a societal level. Although public awareness campaigns about dementia are increasing globally (Alzheimer’s Disease International, 2020b), the education and training standards focused less on this aspect. It is acknowledged that public awareness often forms part of national dementia strategies or action plans.. In line with the Wales Standard (Care Council for Wales, 2016), adopting the concept of standard influencers, who are not health professionals, and promoting specific training for these influencers, could potentially raise the profile of dementia. Influencer learning topics could potentially focus on engagement. Champions are not a new concept and they have been used widely to promote behavior change (Aoun et al., 2013; Shea, 2021). A systematic review concluded that quality improvement champions in nursing homes can increase participation in such projects as well as improve the quality of care, patient outcomes, and job satisfaction (Woo et al., 2017). Although champions and public awareness campaigns have previously been used to change behaviors, we acknowledge it is difficult to determine the efficacy of this approach. It would serve other countries and an international standard to include a training level for influencers and a separate one for the general public.

### Cultural Safety and Rural Issues

Training and education topics focusing on First Nations Peoples were mentioned in four standards and cultural and linguistic backgrounds were listed in five standards. Although cultural safety features were found in some standards, acknowledgment of the cultural and linguistic diversity of the dementia care workforce itself was lacking. Some countries that rely on migrant workers (such as Singapore) already provide dementia education and training for migrant workers (Alzheimer’s Disease International & Alzheimer’s Australia, 2014). An international standard and countries dependent on or using migrant workers should consider adding this element to their standards. Furthermore, countries should also consider the specific needs of First Nations people. This is not adequately addressed in most standards. One U.S. standard (Alzheimer’s Association and Centers for Disease Control and Prevention, 2018) provides an example of how First Nations Peoples can be included in a standard. There was also a lack of focus on rural and remote areas in all standards. In 2021, 43% of the total world population lived in rural areas, with developing countries more likely to have larger proportions of the populations living in rural or remote areas (The World Bank, 2022). Rural and remote areas often have different health care demand and supply issues than their urban-based counterparts (Thomas et al., 2015). It is therefore important to tailor education and training needs to the requirements of both geographical locations and to reflect such needs in a standard.

### Self-Care

Self-care for healthcare professionals is crucial given the impact of job satisfaction on the retention of health and social care workers (Nancarrow et al., 2014), the high rates of burnout among health professionals (Fitzpatrick et al., 2020), and the impact of COVID-19 on workload and constant change in work processes. Indeed, self-care is increasingly heralded as an important element of education and training in medical training colleges and health professional training and education programs. In 2011, a systematic review already stressed the importance of personal development and self-care for care staff (Tsaroucha et al., 2011). More recently, Fitzpatrick et al. (2020) identified that doctors in training who report that their hospital promotes and prioritizes well-being measures, which includes self-care initiatives, are less likely to experience burnout. Although it currently did not feature strongly in existing standards, albeit one standard only, developers should consider including self-care as an essential part of any future dementia education and training standard, especially given the global crisis in health workforce supply.

### Digital Literacy and Health Informatics

Digital literacy for people living with dementia, formal and informal carers, and health informatics was mentioned in only six standards as a training or education topic and thus did not have a strong focus in the existing standards. Given the rapid changes that have occurred in the care of older people such as telehealth implementation due to COVID-19 as demonstrated by Fisk et al. (2020) in the United Kingdom, the United States, and Australia, it is paramount that both digital literacy training and education for people living with dementia, formal and informal carers, and health informatics training are included in future standards.

## Limitations

First, this review focused on the development of standards and not on implementation, such as training delivery models, or curricula of dementia education and training. Our review did look at reported barriers and enablers of standards development; however, we did not include the evaluation of standards implementation.

Second, the findings are not necessarily generalizable to developing countries, where dementia is also rising. Only standards available in English since 2010 and those identified in developed countries were included in this review. An attempt was made to find standards in selected non-English-speaking developed countries, but was not found. Of particular concern is the fact that 60% of people living with dementia are from low- and middle-income countries. This figure is estimated to reach 70% by 2050. Asia has the largest population of people living with dementia and it is expected to increase from 29 million in 2020 to 82 million people in 2050. Although Africa has the smallest population of people living with dementia (5 million in 2020), this number is estimated to at least triple and reach 17 million people in 2050 with variations between countries (Alzheimer’s Disease International, 2020b). For example, Adeloye et al. (2019) conducted a systematic review in 2019 of the prevalence of people living with dementia in Nigeria and found an estimated increase from 63,000 in 1995 to 318,000 in 2015 for people aged 60 years and older which equates to a 400% increase over 20 years. The authors call for policy-makers in Nigeria to ensure that adequate infrastructure, dementia care staff training, and research are developed to improve dementia care. The importance is further highlighted by the ADI that currently, no African country has yet developed a national dementia plan (Alzheimer’s Disease International, 2021b). It is, therefore, imperative to ensure that developing countries are included as key stakeholders when developing dementia training standards.

Third, this scoping review was not focused on research studies themselves but on national or state-based standards and this could be seen as a limitation. However, the majority of the standards were all developed based on existing evidence such as Ireland (S4; Irving et al., 2014), Scotland (S8; The Scottish Government, 2011), and the Australian “Four steps to building Dementia Practice in Primary Care” (S1; Australian Primary Health Care Nurses Association, 2015). Countries that started earlier with tackling dementia on a national scale are also further ahead with robust evaluation plans. In particular, the United States provides a good example of national tracking of their national plan to address Alzheimer’s disease and provides an annual update (US Department of Health and Human Services, 2019).

## International Implication and Future Application

The rising demand for high-quality dementia care staff requires increased transparency to safeguard quality. Simultaneously, there is a strong global push from various international organizations demanding transparency in human capital reporting for internal and external stakeholders (International Organization for Standardization, 2018; World Economic Forum, 2022). Thus, an international standard on dementia education and training would benefit from developing quantitative and qualitative reporting metrics to allow for increased transparency in educating and training the dementia care workforce that can be used to guide governments and organizations to measure success. Further work is needed in collaboration with the international community, especially from developing countries to ensure the applicability of an international standard across regions. Ideally, the International Organization for Standardization Technical Committee Technical Committee 314 Aging Societies can assist in this area. This organization is well placed given its expertise and ability to coordinate and includes large international expert groups (Pit, Livingstone, et al., 2022). Second, the results could also be used by other countries that have yet to develop a national dementia education and training standard. Third, the value of using an international standard to measure sustainable employability in hospitals as demonstrated by (Fitzpatrick et al., 2020) supports the argument that an international standard on dementia education and training could be evaluated in practice to further improve the development of such standards.

## Conclusion

This scoping review was the first step to inform the development of an international standard for country-specific dementia education and training standards. Thirteen standards, published between 2010 and 2020, were identified. The content of the standard varied in complexity in terms of principles, learning topics, training/education levels, selected outcomes, training resources, and recommended strategies. In line with the WHO philosophy of “nothing about us without us,” people living with dementia should be major stakeholders in the development of a standard for dementia education and training. High-quality partnerships, sustainable funding, organizational support for implementing learning into practice, and strong standard implementation and evaluation plans are crucial. Dementia training and education should be part of the larger workforce planning cycles to safeguard the recruitment and retention of the right people to care for people living with dementia, to ensure a high-quality and sustainable dementia workforce.

## Supplementary Material

gnad023_suppl_Supplementary_Material
