# Supplementary material for: International Standards for Dementia Workforce Education and Training: A Scoping Review
Source: Gerontologist. 2023 Apr 18;64(2):gnad023. doi: 10.1093/geront/gnad023 (PMC10825835; doi:10.1093/geront/gnad023)
Supplement: gnad023_suppl_Supplementary_Material [file gnad023_suppl_supplementary_material.docx]

**Online Supplementary Material**

**Section 1: Search strategy (Adapted from Pit et al., 2022a).**

Domains for generating key words: standards/framework, workforce, education/training, dementia

Grey Literature searches: standards/framework/guide for workforce/worker/carer education/training in dementia/Alzheimer’s/cognitive impairment, by country, 2010-2020

Database searches:

Standards/frameworks:

MeSH: Guidelines

Other: standard*, framework, guide, schema, model, benchmark, structure

Workforce

MeSH: Workforce; Health Workforce; Health Personnel; Allied Health Personnel; paramedics; Healthcare Assistants; Healthcare Support Workers; physicians; Physicians, Primary Care; Nurses; nursing staff; Registered Nurses; Nursing Staff, Hospital; Nurses, Community Health; Geriatric Nursing;

Other: Staff, health workers, social workers, psychologists, psychiatrists, nursing staff, placement nurse*, registered nurse*, nurse*, aged care worker, residential support worker, community support worker, assistant in nursing, personal care assistant/associate

Education /Training

MeSH: Vocational Education; Staff Development; Education; Teaching

Other: educating, instruction, learning, training,

Dementia

MeSH: Dementia; Frontotemporal Dementia; Dementia, Multi-Infarct; Alzheimer Disease; Lewy Body Disease; Dementia, Vascular; Neurocognitive Disorders;

Other: Alzheimer’s disease, Cognitive impairment, Memory loss, Alcohol related dementia

Countries of interest

MeSH: Australia, New South Wales, Queensland, Victoria, Western Australia, South Australia, Tasmania, Norther Territory, Australian Capital Territory

New Zealand, Canada

United States (New England, Southeastern United States, Southwestern United States, Northwestern United States, Midwestern United States)

United Kingdom, Scotland, Ireland, Wales

Europe, Netherlands, Germany, Sweden

Scandinavian and Nordic Countries (NORWAY; SWEDEN; DENMARK; ICELAND; FINLAND)

Other: Scandinavia

**Section 2: Table S1. Key sections of Standards (Adapted from Pit et al., 2022a).**

| **Content** | **Standards with this content** |
| --- | --- |
| **Potential elements for introduction of the standard** |  |
| Preamble by person living with dementia | S6 |
| Frequently asked questions | S12 |
| Explanation of how to use the standard | S5, S7, S8, S9, S10, S12 |
| Structure of standard | S1, S2, S3, S4, S5, S6, S8 |
| Evidence supporting the standard (e.g., policy and research) | All |
| Purpose and guiding principles | All |
| Demonstration of the dementia journey | S6, S8 |
| **Potential elements of the main body of the standard** |  |
| Tiers or levels of practice or training: | S3, S4, S5, S6, S7, S8, S9, S13 |
| - population needs and workforce capability | S3 |
| Topics or Priority Areas, which the following elements: | All |
| - Tiers or level of practice or training | S3, S4, S5, S6, S7, S8, S9, S13 |
| - Explanation of context | S6, S9, S10 |
| - Evidence to demonstrate effectiveness | S10 |
| - Quotes and stories by People Living with Dementia | S6, S8 |
| - Case studies | S10 |
| - Key Audience | All |
| - Strategies, actions, or recommendations | S1, S2, S4, S5, S7, S10, S11 |
| - Learning outcomes by level- knowledge | S6, S8, S9 |
| - Learning outcomes by level – skills | S6, S8, S9 |
| - Indicator, Outcome measures, Success factors or Skills statement | S1, S2, S3, S5, S8, S10, S12, S13 |
| - Links to appropriate guidance and/or legislation | S5, S5, S6, S7, S8 |
| - Links to appropriate training resources | S3, S5, S7, S9, S12, S13 |
| **Potential tools listed in standards** |  |
| Self-assessment tool | S6 |
| Workforce Planning Tool | S7 |
| Online database to track all education and training activities | S13 |
| **Potential final key sections for a standard** |  |
| Implementation plan | S1, S2, S3, S4, S5, S7, S8 |
| Evaluation plan | S2, S3, S4, S5, S8, S9, S10, S11 |
| Taxonomy of agreed definitions and principles | S3, S5, S10 |
| Research Recommendations | S5 |
| Explanation of how health professionals can receive payment for services provided | S1 |
| National occupational standards, skills frameworks & regulated qualifications components or care standards are used for standard development | S3, S5, S9 |
